# Susceptibility of monomicrobial or polymicrobial biofilms derived from infected diabetic foot ulcers to topical or systemic antibiotics *in vitro*

**DOI:** 10.1371/journal.pone.0228704

**Published:** 2020-02-18

**Authors:** Bianca L. Price, Robert Morley, Frank L. Bowling, Andrew M. Lovering, Curtis B. Dobson

**Affiliations:** 1 Division of Pharmacy and Optometry, Lydia Becker Institute of Immunology and Inflammation, Faculty of Biology Medicine and Health, University of Manchester, Manchester, United Kingdom; 2 Podiatric Surgery Dept, Buxton Hospital, Derbyshire Community Health Services NHS Foundation Trust, Bakewell, United Kingdom; 3 Division of Diabetes, Endocrinology & Gastroenterology, Faculty of Biology Medicine and Health, University of Manchester, Manchester, United Kingdom; 4 Microbiology Department, Antimicrobial Reference Laboratory, Bristol, United Kingdom; 5 Medical Device Biology Group, Division of Pharmacy and Optometry, Faculty of Biology Medicine and Health (FBMH), University of Manchester, Manchester, United Kingdom; The University of Jordan School of Pharmacy, JORDAN

## Abstract

Diabetic foot ulcers can become chronic and non-healing despite systemic antibiotic treatment. The penetration of systematically-administered antibiotics to the site of infection is uncertain, as is the effectiveness of such levels against polymicrobial biofilms. We have developed an *in vitro* model to study the effectiveness of different treatments for infected diabetic foot ulcers in a wound-like environment and compared the activity of systemic levels of antibiotics with that for topically applied antibiotics released from calcium sulfate beads. This is the first study that has harvested bacteria from diabetic foot infections and recreated similar polymicrobial biofilms to those present *in vivo* for individual subjects. After treatment with levels of gentamicin attained in serum after systemic administration (higher than corresponding tissues concentrations) we measured a 0–2 log reduction in bacterial viability of *P*. *aeruginosa*, *S*. *aureus* or a polymicrobial biofilm. Conversely, addition of gentamicin loaded calcium sulfate beads resulted in 5–9 log reductions in *P*. *aeruginosa*, *S aureus* and polymicrobial biofilms derived from three subjects. We conclude that systemically administered antibiotics are likely to be inadequate for successfully treating these infections, especially given the vastly increased concentrations required to inhibit cells in a biofilm, and that topical antibiotics provide a more effective alternative.

## Introduction

Diabetic foot problems are the most common cause of non-traumatic limb amputation in the UK, with the incidence of diabetes related major amputation at 1.1 per 1000 persons [1,2]. Following amputation there is a 40–50% mortality rate at five years [3]. Up to 25% of people living with diabetes will experience a diabetic foot ulcer (DFU), which have a 40% incidence of recurrence [4–6]. These ulcers often become chronic and infected with bacterial biofilm [7]. Systemic antibiotics are prescribed when an ulcer is showing clinical signs of infection [8–10]. Resolution of infection after treatment of Diabetic Foot Infection (DFI) with systemic antibiotics varies widely with values reported between 5.6% and 77.8% [11].

At a high bacterial load, the biofilm is likely to be very well established and highly tolerant to antibiotics [12,13]. DFIs are typically colonised with bacteria similar to the surrounding skin and become more complex in microbial diversity over time and with progression of the ulcer [14–17]. *Staphylococcus* spp., *Corynebacterium* spp., and *P*. *aeruginosa* are common infectious organisms [18,19] however the organisms associated with these infections are diverse [20,21].

On presentation with an ulcer the patient care pathway involves offloading and debridement [22]. If signs of infection are apparent, patients are prescribed antibiotics orally or intravenously based on local prescribing guidelines [23,24]. Typically, systemic antibiotics are considered to have achieved sufficient concentrations if they reach the minimum inhibitory concentration (MIC) for the target bacteria [25]. However, it is now widely acknowledged that bacteria form biofilms in soft tissue infections most if not all of the time [7]. This is significant because biofilms have substantially increased tolerance to antibiotics, sometimes by a factor of 1000 [12,26,27], and therefore the MIC is not a useful predictor of successful treatment [28]. There is also increasing incidence of antibiotic resistance in bacterial infections, including DFIs [29]. Moreover, antibiotics are often prescribed before the bacteria in the ulcer are identified meaning that the most appropriate antibiotics are not always given in the first instance [20,30,31].

When studying biofilms, investigators often look at lab strains of bacteria in monoculture using readily available substrates in the lab like plastic or agar because these studies can be carefully controlled and the methods and strains are well characterised [32,33], although some polymicrobial models of infection have been developed [34–36]. However, clinical isolates often behave differently to lab strains and there is evidence that polymicrobial biofilms have altered tolerance to antibiotics and form more robust biofilms with different architecture because of symbiotic interactions between the different species [35,37]. Increasingly there is awareness that laboratory biofilm models that use abiotic surfaces as the substrate for biofilm growth do not reflect environmental or *in vivo* biofilms and additionally that factors produced *in vivo* such as growth factors, proteases and serum proteins are absent in traditional microbiological media [38].

We have previously sought to address some of these issues where they apply to wound biofilms by using type 1 collagen, the predominant matrix component in the dermis, as a substrate for biofilm formation [33]. Here we have further developed the model to more accurately reflect the dermis by incorporating hyaluronic acid (HA), extracellular matrix protein extract (ECM) and fibroblasts. Fibroblasts are important in remodelling of the dermal matrix (40) as well as producing secreted factors such as extracellular matrix components, signals and enzymes and therefore fibroblast conditioned media has been used to study the effect of biofilms on wound healing (41). In this study we have applied these insights to develop an *in vitro* matrix (IVM) model relevant to DFI in which we can grow polymicrobial biofilms. We collected samples of superficial infected foot ulcers, grade 1B according to the Wagner scale [39], harvested and identified the bacteria and used these clinical isolates to inoculate the IVM model. As such this is the first study to have specifically studied and recreated the microbiome of superficial infected grade 1B ulcers from single subjects. We then tested gentamicin at the maximum concentration reported in serum (C_max_) and compared to gentamicin released topically from calcium sulfate beads (CSB) (Stimulan, Biocomposites Ltd, UK) to establish the efficacy of gentamicin in each case. This work highlights a potential novel approach to treatment of ulcers where biofilm eradication concentrations cannot be achieved by systemic antibiotics.

## Materials and methods

### Ethics

This study was approved by the HRA and Wales Rec 6 ethics committee and sponsorship approval was obtained from University of Manchester FBMH ethics. Inclusion criteria specified subjects were above the age of 18 who presented with grade 1B DFI according to the Wagner scale and had not received prior treatment with antibiotics for their DFI. Patients were excluded if they were unable to give informed consent. Debrided tissue was stored on ice until collection by the research team for processing on the same day.

### Bacterial strains, media and culture conditions

The laboratory strains used in this study were the Nottingham *Pseudomonas aeruginosa* PA01, donated by Miguel Camara and the *Staphylococcus aureus* ATCC 6538 (LGC standards). Overnight cultures were prepared in tryptic soya broth (Oxoid) and diluted to 10^5^ CFU/ml in fibroblast growth media (FGM) (Dulbecco Modified Eagle Medium (Sigma D5546 low glucose) supplemented with 10% fetal bovine serum (FBS)(Sigma), 80 mM HEPES (MP Biomedicals) and 4 mM glutamine (Sigma)) [40], 500 μl of diluted culture were used to inoculate models with *S*. *aureus* and *P*. *aeruginosa* as previously described [33]. Debrided tissue (DFAN, DFAL, DFAC) was sampled from three different subjects, weighed, and homogenised in a bead beater (Precellys) using glass beads until the tissue had completely homogenised. Bacteria were pelleted from lysates, diluted and plated onto chocolate agar (tryptic soya agar (Oxoid) supplemented with lysed 5% horse blood (Hardy Diagnostics)) and incubated at 37 °C under aerobic, 5% CO_2_ and anaerobic conditions for 72 hours. For inoculation into IVM models, bacteria were harvested directly from agar plates that had been incubated at 5% CO_2_ as these reflected best the diversity of the wounds by emulsifying the colonies in 1 ml phosphate buffered saline (PBS) (Oxoid) using a spreader, then collecting the emulsion and adjusting to an optical density (A_600_) of 1.0 and then diluting to 1×10^−3^. This method was chosen to obtain the closest ratio as possible to that isolated from the sample.

### Preparation of calcium sulfate beads

A 10cc Stimulan Rapid Cure kit (Biocomposites Ltd) contains 20g of pharmceutical grade calcium sulfate hemihydrate powder. To this calcium sulfate, 6 ml of sterile water (for unloaded beads) or 6 ml of a 40 mg/ml gentamicin sulfate solution (Amdipharm) was added and mixed for 30 seconds into a paste under sterile conditions. The paste was pressed into 3 mm diameter hemispherical cavities in a flexible mold and left to set for 10 minutes before beads were pressed out [27].

### Identification of bacterial isolates

Bacterial colonies were counted and examples of each different colony morphology were isolated and restruck to single colonies. Resultant colonies were isolated and transferred to 20 μl of sterile MilliQ water in a PCR tube and and boiled for 10 minutes. This lysate was used as the template for the subsequent PCR reaction. Primers to the 16S rRNA gene 8FLP and 806R were used, as published previously [41]. PCR was conducted using a TGradient thermocycler (Biometra Göttingen, Germany) and run for 35 thermal cycles of 94°C (1 min), 53°C (1 min), and 72°C (1 min). A 15-minute elongation step was included in the final cycle. PCR products were purified using a QIAquick PCR purification kit (Qiagen, West Sussex, UK) as per the manufacturer’s instructions. A reaction mixture containing 4 pM forward or reverse primer and 40 to 50 ng of DNA in a 10 μl total volume was used for DNA sequencing. DNA sequencing was performed using the Applied Biosystems 3730 DNA analysis system at the DNA Sequencing Facility within The University of Manchester.

### Preparation of *in vitro* matrix models

IVM models were adapted from those published previously [40]. We added HA (Alfa Aesar) and ECM (Sigma) so that the matrix represented that of a dermis as closely as possible. These components may be important in the activity of the antibiotics as binding factors as well as influencing the fibroblasts and bacteria in the model [42,43]. Human dermal primary fibroblasts (HDFn, Life Technologies) were maintained as per manufacturer’s instructions except that cells were grown in FGM. Once HDFn reached 80% confluency they were split 9:10 and allowed to grow overnight. The following day cells were harvested by trypsinisation and diluted to 3×10^5^ cells/ml in FGM.

IVM models were prepared in 6-well plates with a transwell insert (3 μm pore size, Corning) in each well. For the acellular layer, type 1 collagen solution (Corning) was diluted to 4 mg/ml in FGM, the pH was adjusted to 7.5 then 1 ml was pipetted into each insert and left to set at room temperature for 30 minutes. To determine the amount of dermis components to incorporate into the model a checkerboard assay with a range of ECM concentrations of 0, 75, 150, 0.300 and 600 μg/ml and HA concentrations of 0, 1, 2 and 3 mg/ml was undertaken (43). It was established that at a final concentration of 4 mg/ml collagen, supplemented with 1 mg/ml HA and 75 μg/ml ECM with 476 μl of the HDFn stock and pH adjusted to 7.5 allowed robust gelation of the matrix, increased concentration of HA and ECM, combined with collagen, prevented the gel from setting. Six mL of cellular collagen was then pipetted on top of the acellular layer and a mold was fitted to the top of the 6 well plate. Collagen polymerised at 37 °C for 1 hour, the mold was removed and 3 ml FGM was added to each well. The models were incubated for five days and then inoculated with bacteria. After inoculation, models were incubated at 37 °C for three days before the addition of gentamicin (Sigma) in media (FGM) at 20 μg/ml (60 μg gentamicin in total), media only (FGM), 400 mg of unloaded CSB or CSB loaded with gentamicin (3.69 mg in total) for a further three days. At the end of the experiment a sterile scalpel was used to section the models, as described previosuly [32]. In brief, models were removed from inserts and placed into petri dishes. They were sectioned by cutting across the model either side of the central void. This section was then divided into 3–5 sections. Sections were weighed and collagenase (MP Biomedicals) was added as described in Price *et al* 2016. Bacterial cells were pelleted, supernatants were shipped to the Antimicrobial Reference Laboratory (Southmead hospital, Westbury-on-Trym, Bristol, BS10 5NB) for antibiotic quantification, and bacteria were resuspended in PBS, diluted and plated onto Tryptic Soya Agar (TSA)(Oxoid) for enumeration. Log reductions were calculated compared to vehicle controls and all experiments were carried out with three replicates.

### Quantitation of antibiotics

Gentamicin was assayed using an Indiko Plus analyzer with the QMS kit for gentamicin (Thermo Scientific Ltd.). The kits were used and validated as detailed by the manufacturers’ instructions, with the lower limits of quantitation at 0.3 g/ml. Intra-assay precision (as assessed by the coefficient of variation of quality-control samples run at the same time) was less than 7%, while inter-assay precision was less than 10%. Samples with concentrations above the calibration range of the assay were diluted in PBS with the results corrected for any dilutions. All *P* values were determined using a paired Student *t* test, and experiments were carried out in triplicate.

### Histology and live dead staining

After incubation with gentamicin or vehicle controls the IVM models were cut into sections, samples were snap frozen in liquid nitrogen, chilled on dry ice and then frozen samples were sectioned to 20 μm using a cryostat (Leica CM3050 S Research Cryostat) and collected on X-tra adhesive slides (Leica). Slides were defrosted and loaded into a tissue processor for hematoxylin and eosin (H&E) staining following the manufacturer’s instructions (Leica ST5010-CV5030). Images were collected on an Olympus BX63 upright microscope using a 20 x objective and captured and white-balanced using a DP80 camera (Olympus) in colour mode through CellSens Dimension v1.16 (Olympus). For live/dead staining, a Live/Dead *Bac*Light bacterial viability kit (ThermoFisher Scientific) was used according to the manufacturers instructions. In brief, component A and component B were mixed in PBS and the sections were submerged in the resultant dye in the dark for 15 minutes before imaging. Images were collected on a Leica M205 FA upright Stereomicroscope using a 5x PlanAPO LWD objective at the equivalent of 64x magnification and captured using a DFC 565FX (Leica) camera through LAS AF v3.1.0.8587software (Leica). Specific band pass filter sets for GFP and mCherry were used to prevent bleed through from one channel to the next. Images were processed and analysed using Fiji ImageJ (http://imagej.net/Fiji/Downloads).

### Scanning Electron Microscopy (SEM)

IVM models or tissue samples were fixed in 2.5% glutaraldehyde (25% stock solution purchased from Alfa Aesar and diluted in PBS) and 4% paraformaldehyde (Sigma) in 0.1 M HEPES (Sigma) at 4 °C overnight. Samples were washed five times for 5 minutes in 0.1 M HEPES, stained in 1% OsO4 (Sigma) in 0.1 M HEPES for 1 hour, and then washed for 5 minutes four times in distilled H2O. Samples were dehydrated in ethanol and then critical point dried and sputter coated with gold-palladium before examination using an FEI Quanta 250FEG scanning electron microscope to image.

### Minimum inhibitory concentration and minimum biofilm eradication concentration assays

MIC assays were carried out according to the CLSI guidelines [44]. In brief, a dilution series was produced from a stock solution of 5 mg/ml gentamicin. The stock antibiotic concentration (1,024 μg/ml) was diluted 1:1 with bacterial overnight culture and sequentially diluted in 96-well plates to produce final antibiotic concentrations between 1 μg/ml and 512 μg/ml in 200 μl volumes. The OD_600_ was read after overnight incubation. Minimal Biofilm Eradication Concentration (MBEC) assays were adapted from the Innovatech protocol. In brief, 200 μl of overnight culture diluted into Tryptic soya broth (TSB) as described above was inoculated into each well of a 96-well plate. A peg lid (Immuno Nunc TSP) was placed onto the 96-well plate and incubated for 24 h at 37°C. After a brief wash, pegs were incubated in gentamicin at 37 °C for 24 h and then washed in PBS before incubation in fresh TSB overnight at 37 °C, followed by measuring the OD_600_.

## Results

### The *in vitro* matrix model resembles soft tissue

**Fig 1:**
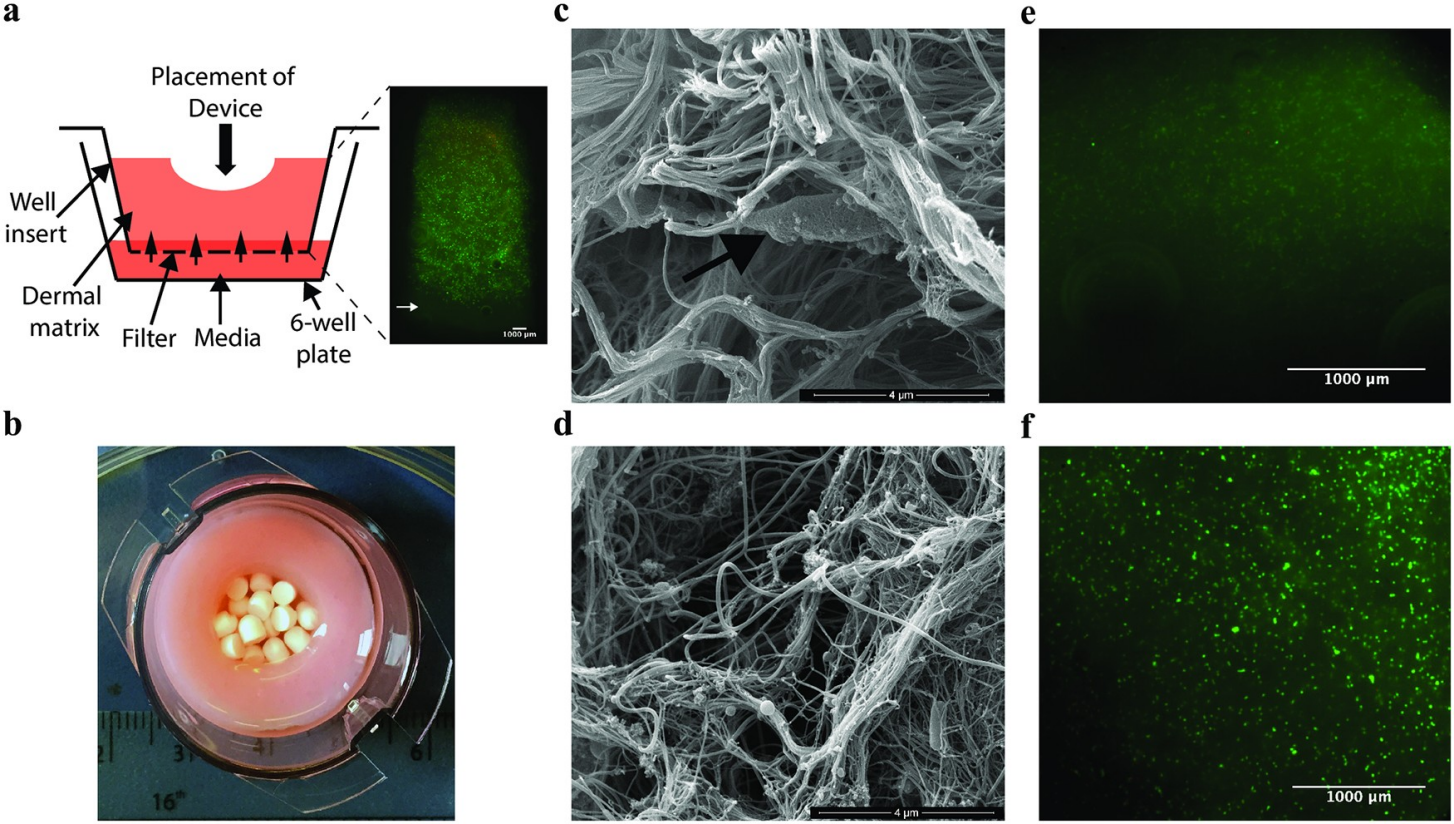
The IVM model system is such that actives can be added to the surface of the model or the media and the matrix is comparable to that of soft tissue. (a) Schematic of the IVM model system and live dead staining of the fibroblasts in cross-section through the model after two weeks maintenance in tissue culture. Green spots indicate live cells, red indicates dead cells, as cells are viable in this image there are very few red spots. (b) Image of the IVM model from above showing the central void packed with CSB with measurements for scale. (c) SEM image of the IVM model with a fibroblast indicated in the collagen matrix by a white arrow (d) compared to DFAN, a sample of debrided tissue. Micrographs of live dead stained sections of representative control models which were not inoculated with bacteria and to which (e) unloaded CSB or (f) gentamicin loaded CSB were added in parallel to inoculated models. There was no evidence of toxicity of the gentamicin or calcium sulfate.

The IVM model was further developed from that previously published [33] by the addition of HA, ECM and fibroblasts into the matrix in the tissue culture insert. The fibroblasts were maintained by the addition of FGM to the well plate surrounding the insert and were viable within control models without inoculation with bacteria for at least two weeks (Fig 1a) [32,33,45]. This configuration allowed us to add gentamicin at the C_max_ concentration to the media at the base of the model in order to simulate delivery of systemic antibiotics, and the CSB loaded with gentamicin as a topical device to the surface of the model (Fig 1a and 1b). Using SEM we visualised the IVM model after three days of biofilm formation and compared these structures with debrided tissue from a diabetic foot infection (Fig 1c and 1d). The synthetic dermal matrix in the model was comparable to that of the debrided tissue.

### Systemic levels of antibiotics are less effective against biofilm than antibiotics released from calcium sulfate beads topically for lab strains grown in monoculture

**Fig 2:**
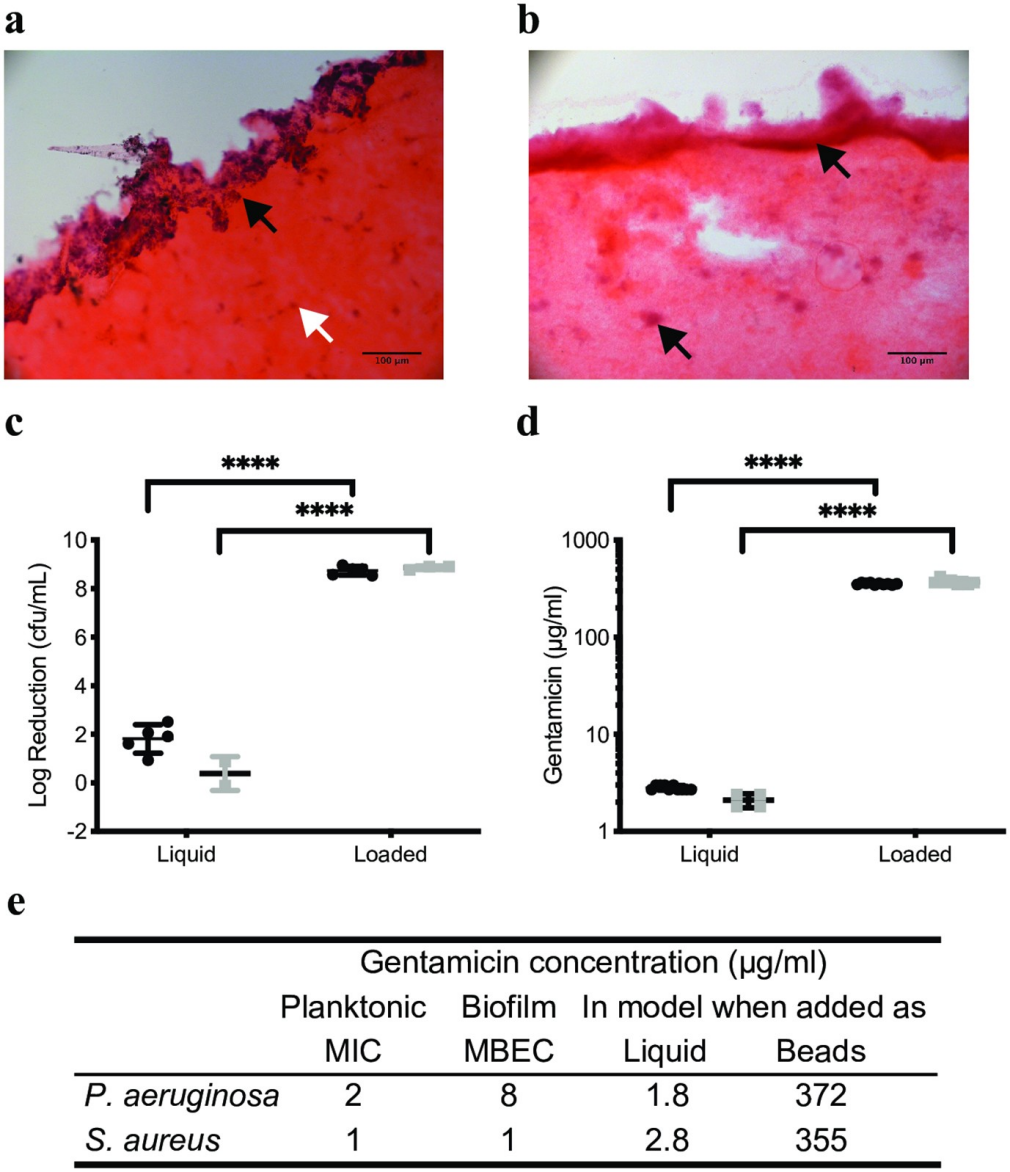
Gentamicin added in CSB eradicated biofilms of *S*. *aureus* and *P*. *aeruginosa* because the amount of gentamicin detected in the bulk of the model exceeded the MIC by two orders of magnitude. The IVM model was sectioned and stained with H&E, biofilm appears purple and the collagen is stained pink. The *S*. *aureus* biofilm (a) localised mostly on the surface of the model (black arrow), dead fibroblasts can be seen in the bulk of the model (white arrow). The *P*. *aeruginosa* biofilm (b) penetrated into the bulk of the model and formed microcolonies (black arrows). (c) Log reductions in *S*. *aureus* (black circles) or *P*. *aeruginosa* (grey squares) were 0–2 when gentamicin at the C_max_ concentration was added to IVM models but exceeded 8 logs when gentamicin loaded CSB were added to the models representing eradication of the biofilm. These log reductions corresponded to concentrations of gentamicin of around 2 μg/ml in the models when liquid gentamicin was added to the models or 350 μg/ml when gentamicin was added in CSB (d). The concentration of gentamicin in the models when added at the C_max_ concentration was around the MIC for *S*. *aureus* and *P*. *aeruginosa* (e), however when added in CSB the concentration of gentamicin in the models was two orders of magnitude higher. **** indicates P<0.0001.

We grew biofilms of laboratory strains of *S*. *aureus* and *P*. *aeruginosa* over 72 hours, which were confirmed by histology using H&E staining, where hematoxylin stains the DNA black and eosin stains the matrix components and cytoplasm pink as done previously (46) (Fig 2a and 2b). We then added gentamicin to the media at the C_max_ of 20 μg/ml [46,47]. This is equivalent to an area under the curve (AUC) concentration of 480 μg/ml which exceeds the target clinical concentration of 70–100 μg/ml for the AUC (Barclay et al. 1995). In parallel we added gentamicin loaded CSB (3.7 (± 0.09) mg of gentamicin), for a further three days (as shown in Fig 1a and 1b). As a result of addition of gentamicin to the media, there was a 1.8 (± 0.6) log reduction in biofilms of *S*. *aureus* but there was no difference in bioburden for the *P*. *aeruginosa* biofilm (0.4 log reduction ± 0.7). (Fig 2c). When gentamicin loaded CSB were added to the models, biofilms of the lab strains *S*. *aureus* and *P*. *aeruginosa* were eradicated (8.7 ± 0.2 and 8.9 ± 0.1 log reductions respectively, P<0.001 for loaded compared to unloaded beads) (Fig 2c). After addition of gentamicin to the media or in loaded CSB gentamicin concentration in the matrix was significantly different at 2.5 (± 0.3) and 386 (± 37) μg/ml respectively (P<0.0001) (Fig 2d).

The MIC and MBEC was established for the lab strains *S*. *aureus* and *P*. *aeruginosa*. Both the MIC and MBEC for *S*. *aureus* were 1 μg/ml. For *P*. *aeruginosa*, a well-known biofilm former, the MIC was 2 μg/ml and the MBEC was 8 μg/ml (Fig 2e). Therefore, when liquid gentamicin was added to the media of the models it exceeded the MIC and MBEC for *S*. *aureus* (liquid gentamicin concentration 2.8 μg/mL; MIC and MBEC 1 μg/ml), although this resulted in only a 1.8 log reduction in the biofilm. For *P*. *aeruginosa* adding liquid gentamicin did not reach the MIC and was over four-fold lower than the MBEC (liquid gentamicin concentration 1.8; MIC 2 μg/ml; MBEC 8 μg/ml).

### Diverse microbiome of a grade 1B ulcer

**Fig 3:**
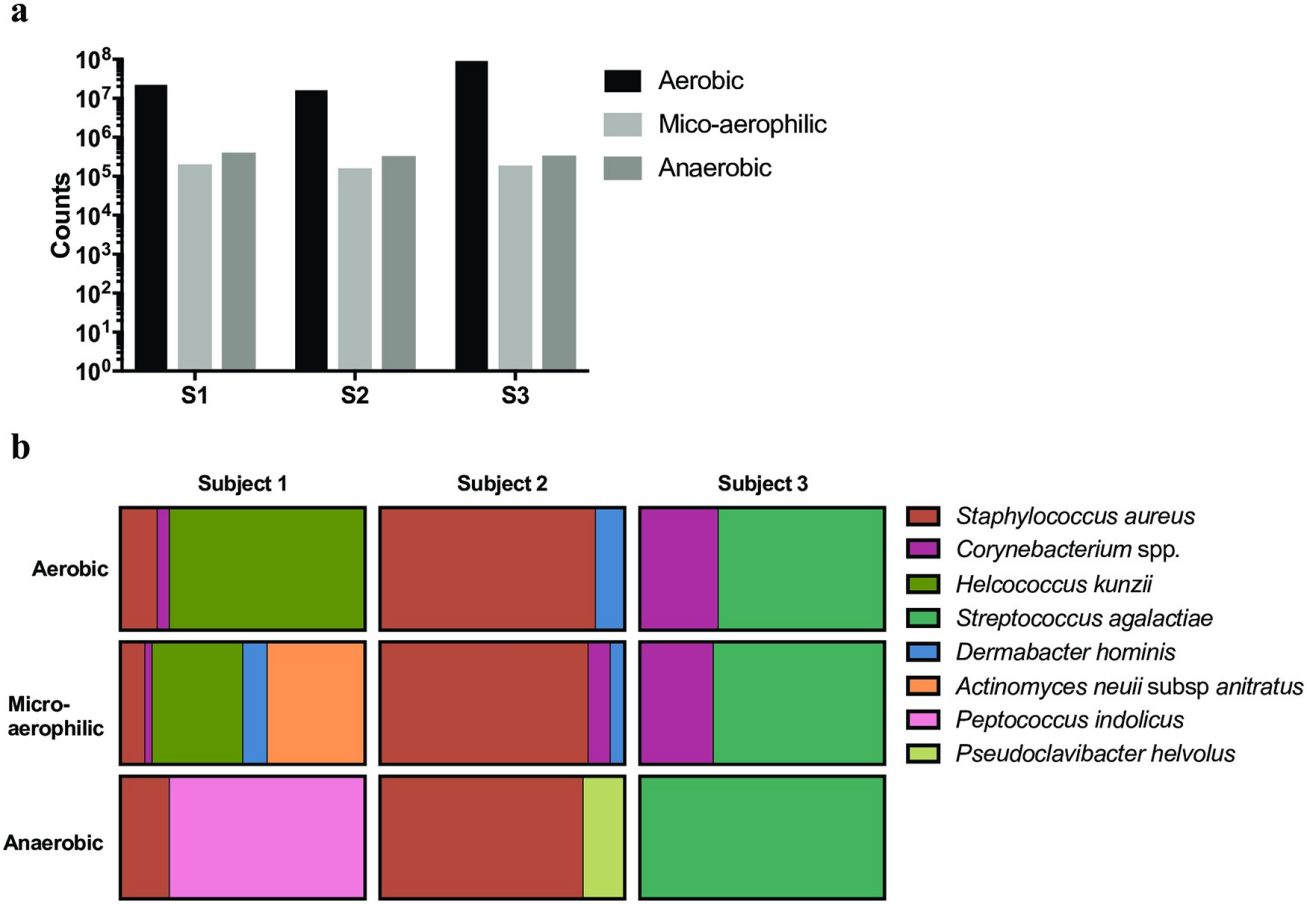
Bacteria were harvested from tissue debrided from DFIs and identified. A sample of debrided tissue was collected from three subjects, homogenised and grown under aerobic, microaerophilic and anaerobic conditions. The counts from each sample are shown in (a). The bacterial identities and proportions for each sample under all three growth conditions are shown as a percentage using parts of whole analysis (b).

We collected debrided tissue from subjects with a DFI designated as grade 1B as identified by the podiatrist. We grew the bacteria under aerobic, micro-aerophilic and anaerobic conditions in order to isolate, proliferate and identify as many different species as possible. The counts for each sample under each condition are shown (Fig 3a). Eight different species of bacteria were identified in total, varying from two to six different strains per subject (Fig 3b). This finding is consistent with data observed by other groups looking at this type of infection [16,17,48]. The culture conditions allowed us to isolate a variety of microorganisms. We found that the microaerophilic condition (5% CO_2_) included all of the species identified under aerobic conditions plus several more and therefore yielded more diverse species than aerobic or anaerobic conditions. Usual skin microbiota of *S*. *aureus* and *Helcococcus kunzii* were identified as well as *Corynebacteria spp* in all the subjects (Fig 3b).

### Exposure of polymicrobial biofilms derived from clinical isolates to gentamicin showed a similar pattern of susceptibility as the lab strains in monoculture

**Fig 4:**
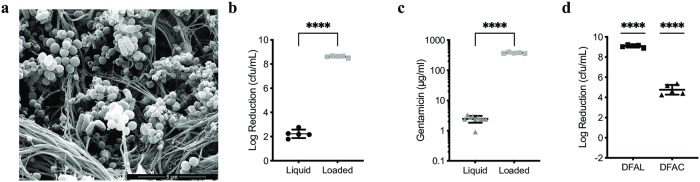
Biofilms were grown in the IVM models using strains isolated from the diabetic foot tissue. These were exposed to either liquid gentamicin and gentamicin loaded CSB or gentamicin loaded CSB only. Increased log reductions were again observed after exposure of biofilms to gentamicin loaded CSB. (a) SEM of a polymicrobial biofilm in the IVM model grown from strains isolated from DFAN. Both rods and cocci can be seen in the matrix. Log reductions in biofilm derived from DFAN upon exposure to gentamicin as a liquid or loaded into CSB were 2 and 8 respectively (P<0.0001), consistent with data in Fig 2(b) and this corresponded to increased gentamicin in the models after exposure to gentamicin in CSB (P<0.0001) (c). Biofilms derived from samples DFAL and DFAC were also grown in IVM models and exposed to gentamicin loaded CSB resulting in eradication of the biofilm or a 5 log reduction respectively (P<0.0001) for gentamicin loaded compared to unloaded beads (d).

Bacteria harvested from debrided tissue were grown on chocolate agar plates and then emulsified directly from the agar plates incubated microaerophilically as this reflected the greatest species diversity, diluted and used to inoculate the IVM models. Biofilms were allowed to develop over three days and imaged by SEM. Shown is a biofilm in the IVM model derived from isolates from sample DFAN (Fig 4a). It is comprised both of rods and cocci and was therefore polymicrobial. The previous experiment comparing liquid gentamicin at the C_max_ and gentamicin loaded CSB was repeated for the polymicrobial biofilm derived from sample DFAN (Fig 4b and 4c).

Liquid gentamicin concentrations available within the IVM model equate to 2.4 ± 0.6 μg/ml and elicited a 2.2 (± 0.4) log reduction in biofilm. Gentamicin concentrations from the gentamicin loaded CSB equate to 377 ± 18 μg/ml and elicited an 8.6 (± 0.1) log reduction in biofilm (Fig 4b). Log reductions after exposure to liquid gentamicin or gentamicin loaded CSB are significantly different, as are concentrations of gentamicin in the models after exposure to gentamicin as a liquid or loaded into CSB (P<0.0001).

Addition of gentamicin loaded or unloaded CSB to biofilms in the IVM models was repeated for the bacteria harvested from samples DFAL and DFAC. After exposure to gentamicin loaded CSB, the biofilm derived from the bacteria from sample DFAL was eradicated (9.1 ± 0.1 log reduction) and a 4.8 (± 0.5) log reduction in biofilm was observed for bacteria harvested from DFAC. P-values of P<0.0001 were calculated for gentamicin loaded beads compared to an unloaded control (Fig 4d).

## Discussion

Our study has shown that supra-MIC concentrations of gentamicin delivered via the bulk fluid are insufficient to inhibit biofilms of *S*. *aureus* and *P*. *aeruginosa* in a wound-like environment because these biofilms have increased tolerance to gentamicin. However, sustained release of high concentrations of gentamicin from calcium sulfate beads in close proximity to the biofilm was effective in reducing biofilm. We have shown previously that concentrations of 2.3 mg/ml gentamicin in similar models is achievable using loaded CSB. In this study we were able to eradicate biofilms of *S*. *aureus* and *P*. *aeruginosa* lab strains as well as polymicrobial biofilms derived from two different subjects, and substantially inhibit biofilm derived from a third subject using gentamicin loaded CSB. The reduced log reductions in the third subject are likely a result of the different species present in the biofilm, *S*. *agalactiae* dominated this biofilm *in vivo* and is known to be resistant to many antibiotics including gentamicin [19,49,50]. The concentration of systemic antibiotics reaching soft tissue after administration has been poorly studied and so little data is available on the efficiency of delivery to the site of infection. One study reports that vancomycin penetration to soft tissue is reduced in diabetics [51]. Conversely, several have shown antibiotics do penetrate into soft tissue in diabetic patients, but this depends on the antibiotic tested [52–54]. Peripheral arterial disease (PAD) is a known risk factor in chronic, non-healing wounds because of reduced oxygenation and immune involvement at the site of infection [55,56], however studies of antibiotic concentrations in DFIs have frequently excluded those with PAD [57] and so the concentration of antibiotics reaching tissue in these patients may be even lower.

In DFIs, successful treatment of wounds may be related to destabilisation of the wound microbiome, rather than complete eradication of the biofilm, and a dynamic biofilm has been suggested to be a positive predictor of healing [15,17]. However, low concentrations of antibiotics are more likely to cause antibiotic resistance and may exert a selection pressure for more virulent microbes [58,59]. Furthermore, the penetration of antibiotics to soft tissue is highly variable between different patients.

Models of infection and biofilms are limited, particularly *in vitro* models [38,60]. The model presented in this study incorporates type 1 collagen, HA, ECM and fibroblasts in order to create a matrix that represents the human dermis as a substrate for biofilm formation. The fibroblasts in the model die rapidly after inoculation with bacteria because of the resultant reduction in pH as well as the toxins produced by the bacteria themselves as seen in previous studies (62). However, the effect of the fibroblasts on the matrix such as matrix contraction and reorganisation, as well as the factors such as proteases present in the conditioned medium have been shown to influence biofilm and therefore were included in this study (63). The model lacks a keratinocyte layer on the surface as well as white blood cells. As a result, the IVM model is unable to model an innate immune system, which may contribute to the higher cell densities seen in the *in vitro* model in this study compared to *in vivo* wounds. The highly enriched composition of the media used in the model is also a likely contributing factor.

In this study we added the C_max_ concentration of gentamicin as a single “dose” because in our closed system the gentamicin would not be lost through excretion. This single dose, reflective of antibiotic levels available in serum, is likely to be a much higher concentration than the amount of gentamicin that is available in soft tissue *in vivo*. If we had access to data on the antibiotic concentration in soft tissue, and added this concentration of gentamicin to the models it is possible that we may not have seen any inhibition of biofilm. The issue is not solely that antibiotics don’t work against biofilms but that they can’t be delivered in sufficient concentrations in a way that is not toxic to the patient. Therefore, for these patients, topical release of antibiotics may provide a viable means to treat infection.

One potential concern with local release of high concentrations of antibiotics from CSB is the possibility of adverse reactions. An assessment of the impact of topical antimicrobials suggested that the risk of an adverse event is probably similar when comparing systemic antibiotics to topical antimicrobial treatments [61]. A recent *in vivo* study with calcium sulfate loaded with vancomycin and tobramycin showed that no vancomycin was detected in the bloodstream and a transient peak of 0.7 μg/ml tobramycin was detected in blood. No inflammatory response was detected in response to the calcium sulfate beads with or without antibiotics [62]. Furthermore, a case-control and cohort study concluded that calcium sulfate void fillers are not a risk factor in nephrotoxicity [63].

Although the use of gentamicin loaded CSB has proved effective in this study, the sample size is small and therefore further work is needed to show that this approach can be effective *in vivo*. Nonetheless further support for the efficacy of such approaches has been reported by case studies [64,65]. In future work it would be useful to add the same concentration of antibiotics to bulk fluid and CSB and calculate log reductions in order to distinguish between the importance of the concentration of antibiotics and the method of delivery.

In conclusion the use of antibiotic loaded CSB provides increased antibiotic concentrations at the site of biofilm growth, compared to those achieved by modelling systemic antibiotic delivery, and is more effective at reducing biofilm populations within an *in vitro* matrix model. CSBs provide a viable means to treat DFI and further investigation is warranted.
